# DcH3.3 and DcNAC1 Regulate the Expression of *UGT73A93* Involved in the Changes in Flower Colour and Fungal Resistance in Carnation

**DOI:** 10.1111/pbi.70674

**Published:** 2026-04-25

**Authors:** Xuhong Zhou, Qirui Xiong, Yue Zhang, Xiumei Yang, Siyuan Zhang, Yanxia Tao, Xiaomi Yang

**Affiliations:** ^1^ Office of Science and Technology Yunnan University of Chinese Medicine Kunming China; ^2^ School of Chinese Materia Medica Yunnan University of Chinese Medicine Kunming China; ^3^ Flower Research Institute Yunnan Academy of Agricultural Sciences Kunming China

**Keywords:** AlphaFold 3, DcH3.3, DcNAC1, *Dianthus caryophyllus*, glucosyltransferase, kaempferol glycosides

## Abstract

Carnation ( *Dianthus caryophyllus* ) contains abundant flavonoid glycosides (FGs), which are important natural functional and colour components. However, there are few reports on the modification of UDP‐glycosyltransferases (UGTs) in relation to flavonoids in carnation. In this study, we cloned and characterised a flavonoid 3′*‐O‐*glucosyltransferase (*UGT73A93*) in carnation in vitro. Overexpression of *UGT73A93* in carnation and tobacco increased flavonoid glycoside accumulation, particularly kaempferol glycosides, while decreasing anthocyanin content and lightening flower colour. *UGT73A93* also enhanced fungal resistance, antioxidant capacity, anti‐amylase and anti‐pancreatic lipase activities. Yeast one‐hybrid and dual‐luciferase assays revealed that the *UGT73A93* promoter interacted with DcH3.3 and DcNAC1, key regulatory proteins involved in flavonoid biosynthesis. We predicted the interaction between DcLON2 and DcNAC1 using AlphaFold 3 and confirmed this hypothesis through yeast two‐hybrid assay and bimolecular fluorescence complementation assays. These findings suggest an epigenetic‐transcriptional cascade (DcH3.3–DcNAC1–UGT73A93) wherein DcH3.3 opens chromatin for DcNAC1‐mediated UGT73A93 activation, while DcLON2 potentially degrades DcNAC1 to form a feedback loop. These results provide new insights into flavonoid 3′*‐O‐*glucosyltransferase and may contribute to future strategies aimed at improving the benefits of flavonoid biosynthesis for both plants and humans. It also demonstrates that AI can be applied in the field of plant biosynthesis, accelerating the process of plant breeding.

## 1 Introduction

Carnation ( *Dianthus caryophyllus* L.), belonging to the family Caryophyllaceae, is an important commercial cut flower worldwide. The primary use of carnation flowers is as vase flowers, with annual global trade valued at approximately USD 25 billion (Filgueira‐Duarte et al. 2024). Carnation cultivars exhibit a wide range of flower colours and patterns, which can significantly impact the flower market economically (Morimoto et al. 2021). Each carnation cultivar typically has a dominant anthocyanin that determines its characteristic colour, such as vermilion, pink, dark red or red purple. The pigment responsible for the deep yellow coloration in carnation flowers is chalcone 2′*‐O‐*glucoside. Additionally, six flavonols—kaempferol 3*‐O‐*rhamnosyl‐(1–2)‐glucoside, kaempferol 3*‐O‐*glucosyl‐(1–2)‐glucoside, kaempferol 3*‐O‐*rhamnosyl‐(1–6)‐glucoside, kaempferol 3*‐O‐*glucosyl‐(1–2)‐[rhamnosyl‐(1–6)]‐glucoside and its malyl ester and kaempferol 3*‐O‐*rhamnosyl‐(1–2)‐[rhamnosyl‐(1–6)]‐glucoside—have been identified as key pigments responsible for cream‐white coloration (Nakayama 2020). Therefore, flavonoids and anthocyanins play crucial roles in the formation of carnation flower colour.

Flavonoids are dominant secondary metabolites in carnations (Nakayama 2020) and play crucial roles in defence against various biotic stresses, such as *Fusarium oxysporum* f. sp. *dianthi* (FOD) (Ardila et al. 2013; Pérez Mora et al. 2021). Additionally, flavonoids exhibit diverse bioactivities with implications for the prevention and treatment of diseases, including ischemic stroke incidence, diabetes and cardiometabolic diseases (Bondonno et al. 2021; Li et al. 2024). Flavonoid aglycones are typically unstable in plant cells and are often modified, such as through glycosylation (Ren et al. 2025). Glycosylation is a key factor contributing to the structural diversity of flavonoids (Alseekh et al. 2020). UDP‐dependent glycosyltransferases (UGTs) are enzymes that catalyse glycosylation reactions in organisms. They transfer sugar moieties from activated donor molecules, such as UDP‐glucose, UDP‐galactose and UDP‐rhamnose, to sugar acceptors, leading to the formation of glycosides (Vogt and Jones 2000). The diverse aglycones and sugar donors necessitate the existence of a large UGT gene family in plants. The flavonoid 3′*‐O‐*glucosyltransferase in plants is related to the flower colour, as well as with pathogen responses (Sharma et al. 2024). Additionally, several transcription factor families, including AP2/ERFBP, bZIP, DREB, WRKY, MYB and NAC, play major roles in abiotic stress responses (Sharma et al. 2024).

The full‐length cDNA clones for 18 glucosyltransferase genes were isolated from petal tissue of carnation (Ogata et al. 2004). The proteins encoded by DcGT4 ( *Dianthus caryophyllus* glucosyltransferase 4) and DcGT5 ( *Dianthus caryophyllus* glucosyltransferase 5), produced in *Escherichia coli* ( *E. coli* ), exhibited broad substrate specificity. These recombinant proteins showed glucosylation activity in vitro, not only with chalcone but also with naringenin, apigenin, kaempferol, quercetin and cyanidin. Both DcGT4 and DcGT5 could transfer glucose to the 7‐OH position of these compounds, as well as to the 3‐OH position of kaempferol and quercetin (Ogata et al. 2004). Although UGTs involved in flavonoid glycosylation have been identified in carnation, the functional characterization of these enzymes, particularly those involved in the biosynthesis of complex flavonoid structures, remains challenging. The catalytic activity of DcGT4 has been studied only in vitro, where it catalyses the synthesis of flavonoid monoglycosides, without involving disaccharides or trisaccharides (Ogata et al. 2004). The true catalytic potential of DcGT4 in plants remains relatively unexplored, despite the fact that such modifications generate considerable chemical diversity and are essential for the stable accumulation of flavonoids.

To confirm whether the enzymes encoded by DcGT4 catalyse the synthesis of flavonoid glycosides from flavonols in vivo, the DcGT4 cDNA will be introduced into plant expression vectors and transferred into tobacco and carnation. This will allow for the exploration of the DcGT4 gene's function. The transcription factor associated with DcGT4 was identified using yeast one‐hybrid technology. Finally, the results will help identify functional plant UGTs for optimising carnation flower colour and resistance, as well as for producing high‐value flavonoid glycosides through synthetic biotechnology.

## 2 Results

### 2.1 Isolation and Expression Level of *UGT73A93*

A full‐length *DcGT4*‐like cDNA was obtained from carnation buds (GenBank accession number OR513509). Following the nomenclature assignment by the International UGT Nomenclature Committee (https://labs.wsu.edu/ugt/submission‐of‐ugt‐sequences‐for‐naming/), this gene is designated *UGT73A93* (*DcGT4*). The predicted open reading frame of *UGT73A93* is 1425 bp, encoding a UGT73A93 protein comprising 475 amino acids.

**FIGURE 1:**
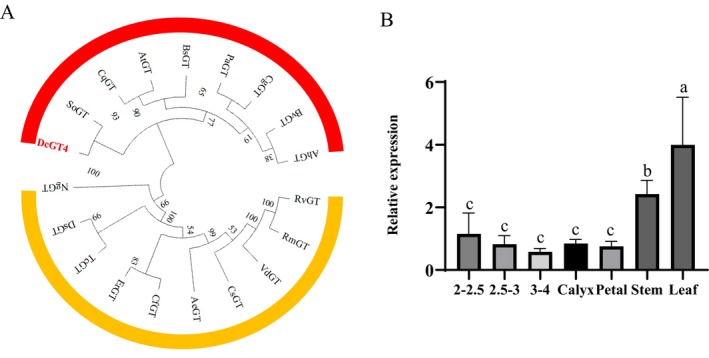
Identification of putative UGT. (A) Phylogenetic analysis of UGT in 19 genea. The numbers on the major branches indicate Bootstrap estimates for 1000 replicate analyses. (B) Expression pattern of *UGT73A93* (*DcGT4*) in different tissues. Error bars represent SE from three biological replicates. Different lowercase letters are significantly different at the 0.05 level of probability. 2–2.5, 2–2.5 cm length of the bud; 2.5–3, 2.5–3 cm length of the bud; 3–4, 3–4 cm length of the bud.

Phylogenetic analyses were performed on 19 genera using their UGT protein sequences (Table S2). The 19 UGT were distributed across two subfamilies (Figure 1A). *UGT* sequences from *Dianthus caryophyllus* and *Saponaria officinalis* clustered in the same branch, suggesting a close phylogenetic relationship between the two genes. *UGT73A93* was mainly expressed in the stem and leaf, with the expression level in young buds being higher than that in large buds (Figure 1B).

### 2.2 Confirmation of *UGT73A93* Function Using Transgenic Tobacco

**FIGURE 2:**
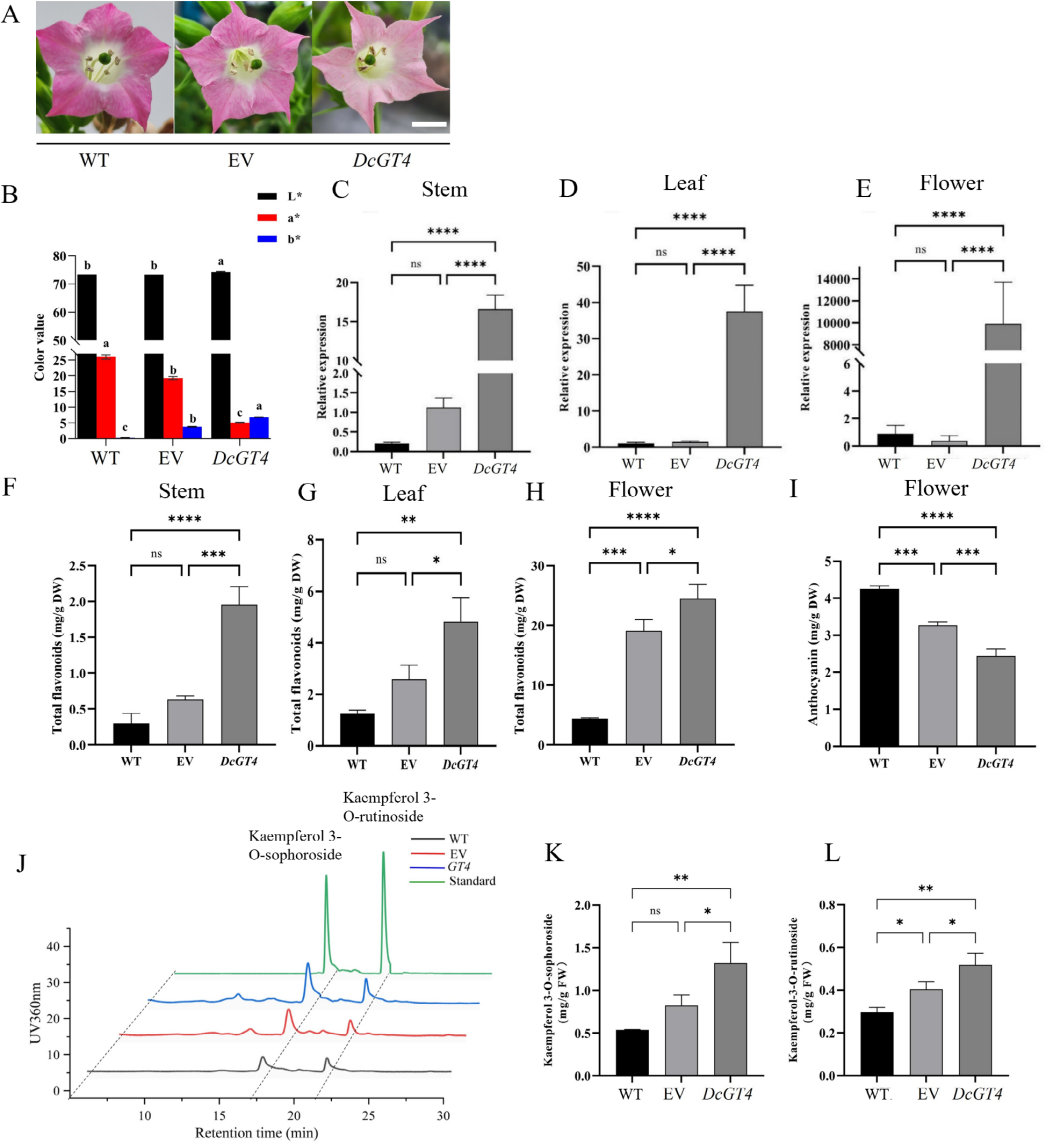
Functional characterization of *UGT73A93* (*DcGT4*) gene following its overexpression in transgenic tobacco lines (T1). (A) Differences in colour between wild‐type (WT), empty vector (EV) and transgenic tobacco flowers. (B) The values of L*, a* and b* classified by the CIELAB systems. (C–E) Expression of *UGT73A93* normalised to *NbGAPDH* gene in WT, EV and transgenic lines of stem (C), leaf (D) and flower (E). (F–H) Contents of the total flavonoids in stem (F), leaf (G) and flower (H). (I) Contents of the total anthocyanins in flower. (J) HPLC chromatograms of flavonols glucosylated in vitro. (K, L) Contents of Kaempferol 3*‐O‐*sophoroside and Kaempferol 3*‐O‐*rutinoside in flower. Error bars represent ±SD from three replicates. Asterisks indicate significant differences by multiple *t*‐test (ns, *p* > 0.05; *p < 0.05; **p < 0.01; ***p < 0.001; ****p < 0.0001). Scale bars represent 1 cm.

To determine the effect of *UGT73A93* on flavonoid biosynthesis, the cDNA of *UGT73A93* under the control of the CaMV 35S promoter was transformed into tobacco plants with red flowers. One independent transgenic plant was generated, showing a flower colour change from red to light red (Figure 2A,B). RT‐PCR analysis confirmed the expression of *UGT73A93* in the T1 generation transgenic lines (Figure 2C–E). The gene expression level was increased by 15–26 567‐fold in plants transformed with the *UGT73A93* overexpression construct, compared to wild‐type (WT) or empty vector (EV) controls. Notably, *UGT73A93* expression levels were significantly higher in the flowers of plants overexpressing the *UGT73A93* gene (Figure 2E).

L* is a measure of the lightness or darkness of a colour, ranging from black to white. The a* and b* components indicate different colour directions in the colour system: a* ranges from red to green and b* ranges from yellow to blue. In the red flowers of WT, EV and *UGT73A93*, the lightness component (L*) of the petals ranged around 74, while the chromatic components a* and b* had values in the ranges of 5–26 and 0.25–6.82, respectively (Figure 2A,B). The average a* value decreased by 80.50% in *UGT73A93* compared to WT and by 73.68% compared to EV. The average b* value increased by 27.28‐fold in *UGT73A93* compared to WT and by 1.72‐fold compared to EV. A noticeable flower colour difference was observed between the overexpressing lines and WT or EV (Figure 2A).

Compared to the stem, leaf and flower extracts of WT and EV, the total flavonoid content was significantly increased, while the total anthocyanin content was significantly reduced in the *UGT73A93* T1 generation transgenic lines (Figure 2F–I). Correspondingly, the flower's red value decreased and the yellow value increased, which were consistent with the changes in total flavonoid and anthocyanin accumulation. Moreover, the petals of the *UGT73A93* transgenic plants accumulated significantly larger amounts of kaempferol derivatives (kaempferol 3*‐O‐*rutinoside and kaempferol 3*‐O‐*sophoroside) compared to the WT and EV plants (Figure 2J–L). Similar results were observed in T3 generation transgenic tobacco plants. Overexpression of the *UGT73A93* construct resulted in lighter flower colour, increased flavonoid content, decreased anthocyanin content and higher accumulation of kaempferol 3*‐O‐*rutinoside and kaempferol 3*‐O‐*sophoroside compared with WT and EV lines (Figure S1). These results indicate that *UGT73A93* promotes the production of flavonol glycosides by increasing the levels of kaempferol 3*‐O‐*rutinoside and kaempferol 3*‐O‐*sophoroside in tobacco. In conclusion, the ectopic expression of *UGT73A93* in tobacco alters flower colour by modulating flavonoid and anthocyanin biosynthesis.

### 2.3 Antioxidant Capacity of *UGT73A93* , WT and EV Tobacco

DPPH, ABTS free radical scavenging capacity and FRAP were used to assess antioxidant activity. The DPPH radical and ABTS radical cation scavenging activities of WT, EV and *UGT73A93* T1 generation transgenic tobacco stems, leaves and flowers are shown in Table 1. The average DPPH IC_50_ values of the WT, EV and *UGT73A93* tobacco flowers were 69.31, 43.53 and 34.87 μg/mL, respectively. The average ABTS IC_50_ values of the WT, EV and *UGT73A93* tobacco flowers were 3.12, 2.84 and 1.86 μg/mL, respectively. The DPPH and ABTS IC_50_ values for *UGT73A93* tobacco flowers were lower than those of WT or EV, indicating stronger antioxidant activity. Notably, the FRAP assay showed results consistent with the free radical scavenging capacity (Figure S2). As observed in the T1 generation, stems, leaves and flowers of T3 homozygous transgenic lines exhibited higher antioxidant capacity than WT and EV, as determined by DPPH, ABTS and FRAP assays (Figure S3 and Table S5).

**TABLE 1:** Antioxidant activity of total flavonoids in different parts of T1 generation transgenic tobacco.

Tissues	Samples	DPPH (IC_50_ μg/mL)	ABTS (IC_50_ μg/mL)
	WT	273.97 ± 5.26^b^	168.17 ± 7.00^a^
Stems	EV	288.97 ± 5.66^a^	127.80 ± 0.62^b^
	*UGT73A93*	233.50 ± 3.44^c^	124.47 ± 3.24^b^
	WT	193.53 ± 2.58^a^	151.37 ± 9.86^b^
Leaves	EV	113.12 ± 3.75^b^	185.23 ± 3.93^a^
	*UGT73A93*	76.85 ± 2.36^c^	90.78 ± 2.47^c^
	WT	69.31 ± 0.65^a^	3.12 ± 2.51^a^
Flowers	EV	43.53 ± 1.52^b^	2.84 ± 1.05^a^
	*UGT73A93*	34.87 ± 4.48^c^	1.86 ± 0.12^a^

*Note:* Means with different lowercase letters within the same column are significantly different at the 0.05 level of probability.

All experimental results showed that flowers exhibited the highest antioxidant capacity of T1 and T3 generation transgenic lines compared to stems or leaves across all three antioxidant assays. Therefore, overexpression of *UGT73A93* increased the antioxidant capacity of tobacco.

### 2.4 *UGT73A93* as a Key Gene for Boosted Flavonoid Glycosides Biosynthesis in Carnation

**FIGURE 3:**
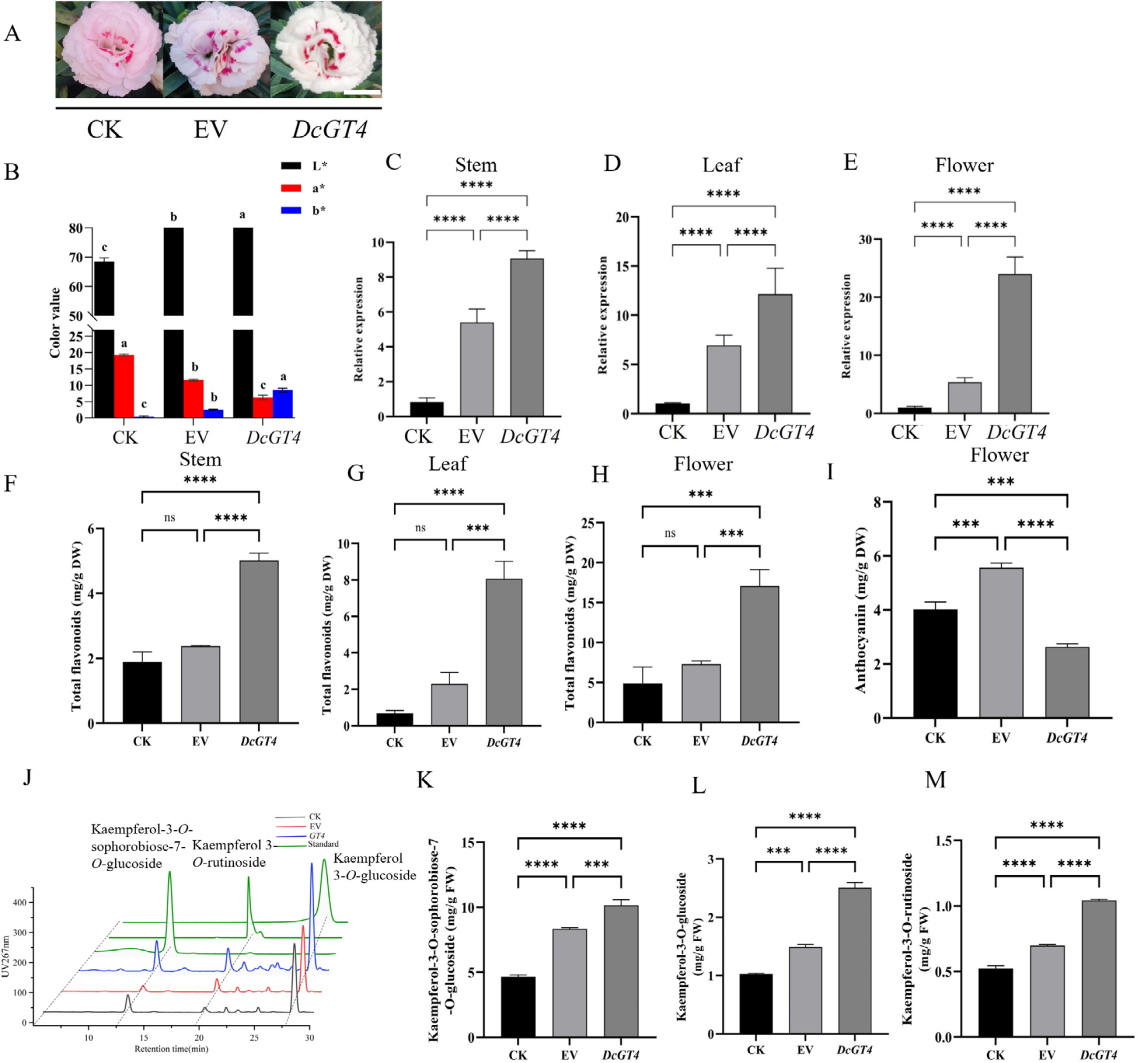
Functional characterization of *UGT73A93* gene following its overexpression in transgenic carnation lines. (A) Differences in colour between wild‐type (WT), empty vector (EV) and transgenic carnation flowers. (B) The values of L*, a* and b* classified by the CIELAB systems. (C–E) Expression analysis of the *UGT73A93* (*DcGT4*) in WT, EV and transgenic lines of stem (C), leaf (D) and flower (E). (F–H) Contents of the total flavonoids in stem (F), leaf (G) and flower (H). (I) Contents of the total anthocyanins in flower. (J) HPLC chromatograms of flavonols glucosylated in vitro. (K–M) Contents of Kaempferol‐3*‐O‐*sophorobiose‐7*‐O‐*glucoside, Kaempferol 3*‐O‐*rutinoside and Kaempferol 3*‐O‐*glucoside in flower. Error bars represent ± SD from three replicates. Asterisks indicate significant differences by multiple *t*‐test (ns, *p* > 0.05; ***p < 0.001; ****p < 0.0001). Scale bars represent 1 cm.

Transgenic carnations overexpressing *UGT73A93* exhibited altered flower colour, resulting in lighter flowers. Overexpression of *UGT73A93* reduced the intensity of the plant's pink petal colour (Figure 3A,B). The average a* values decreased by 67.51% in *UGT73A93* compared to WT and by 45.95% compared to EV. The average b* values increased by 21.67‐fold in *UGT73A93* compared to WT and by 3.34‐fold compared to EV. A clear flower colour difference was observed between the overexpressing lines and the WT or EV (Figure 3A). We analysed the expression levels of the transgene at the mRNA level in carnation stems, leaves and flowers (Figure 3C–E). The gene expression level was increased by 1.20–11.36‐fold in the plants transformed with *UGT73A93* compared to WT and EV. Notably, in flowers, *UGT73A93* expression was highest in the overexpression *UGT73A93* transgenic plants compared to WT and EV (Figure 3E).

The overexpression of *UGT73A93* promoted the total flavonoid content (TFC). *UGT73A93* significantly affected the TFC in different tissues of carnation (Figure 3F–H). Additionally, the overexpression of the *UGT73A93* gene was associated with a decrease in total anthocyanin content in carnation flowers (Figure 3I). Compared to WT and EV lines, the contents of kaempferol‐3*‐O‐*sophorobiose‐7*‐O‐*glucoside, kaempferol 3*‐O‐*rutinoside and kaempferol 3*‐O‐*glucoside in flowers were significantly higher in the *UGT73A93* transgenic lines (Figure 3K–M). These results suggest that *UGT73A93* may be a promising gene for improving kaempferol glycoside production in carnation flowers.

To investigate the coordinate interaction of the *UGT73A93* gene with other flavonoid biosynthetic genes in transgenic carnation, the expression levels of structural genes such as *DcFLS*, *DcGT5*, *DcF3′H* and *DcDFR* were analysed by real‐time qPCR. Among these, *DcFLS*, *DcGT5*, *DcF3′H* and *DcDFR* were consistently downregulated in the UGT73A93 overexpression lines compared to WT and EV lines (Figure S4). These results suggest that overexpression of *UGT73A93* may modulate the expression of key structural genes in the flavonoid biosynthetic pathway.

### 2.5 Antioxidant Activity of *UGT73A93* , WT and EV Carnation

In this study, DPPH, ABTS and FRAP assays were conducted to evaluate the antioxidant activities of plants in vitro (Table 2). In DPPH assay, the average DPPH IC_50_ values ranged from 58.59 to 171.97 μg/mL. The DPPH IC_50_ values of *UGT73A93* were lower compared to those of WT and EV in different tissues. In the ABTS assay, the average ABTS IC_50_ values ranged from 16.94 to 95.06 μg/mL. *UGT73A93* also showed lower ABTS IC_50_ values compared to WT and EV across different tissues. In the FRAP assay, *UGT73A93* exhibited higher values at 1000 μg/mL extracts compared to WT and EV in all tissues (Figure S5). Notably, the antioxidant activities were the highest in the flowers compared to other plant tissues. Therefore, overexpression of *UGT73A93* enhanced the antioxidant capacity of carnation.

**TABLE 2:** Antioxidant activity of total flavonoids in different parts of transgenic carnation.

Tissues	Samples	DPPH (IC_50_ μg/mL)	ABTS (IC_50_ μg/mL)
	WT	171.40 ± 3.54^a^	35.41 ± 0.76^b^
Stems	EV	171.97 ± 8.03^a^	95.06 ± 10.72^a^
	*UGT73A93*	163.60 ± 7.41^a^	29.71 ± 5.87^b^
	WT	118.93 ± 5.99^a^	37.86 ± 4.39^b^
Leaves	EV	72.39 ± 4.28^b^	65.90 ± 7.84^a^
	*UGT73A93*	68.64 ± 1.44^b^	32.00 ± 1.35^b^
	WT	93.38 ± 3.72^a^	28.59 ± 4.93^a^
Flowers	EV	62.75 ± 3.14^b^	23.48 ± 4.52^ab^
	*UGT73A93*	58.59 ± 2.92^b^	16.94 ± 1.37^c^

*Note:* Means with different lowercase letters within the same column are significantly different at the 0.05 level of probability.

### 2.6 Enzyme Inhibition and Fungal Inhibition in *UGT73A93* , WT and EV Carnation

**FIGURE 4:**
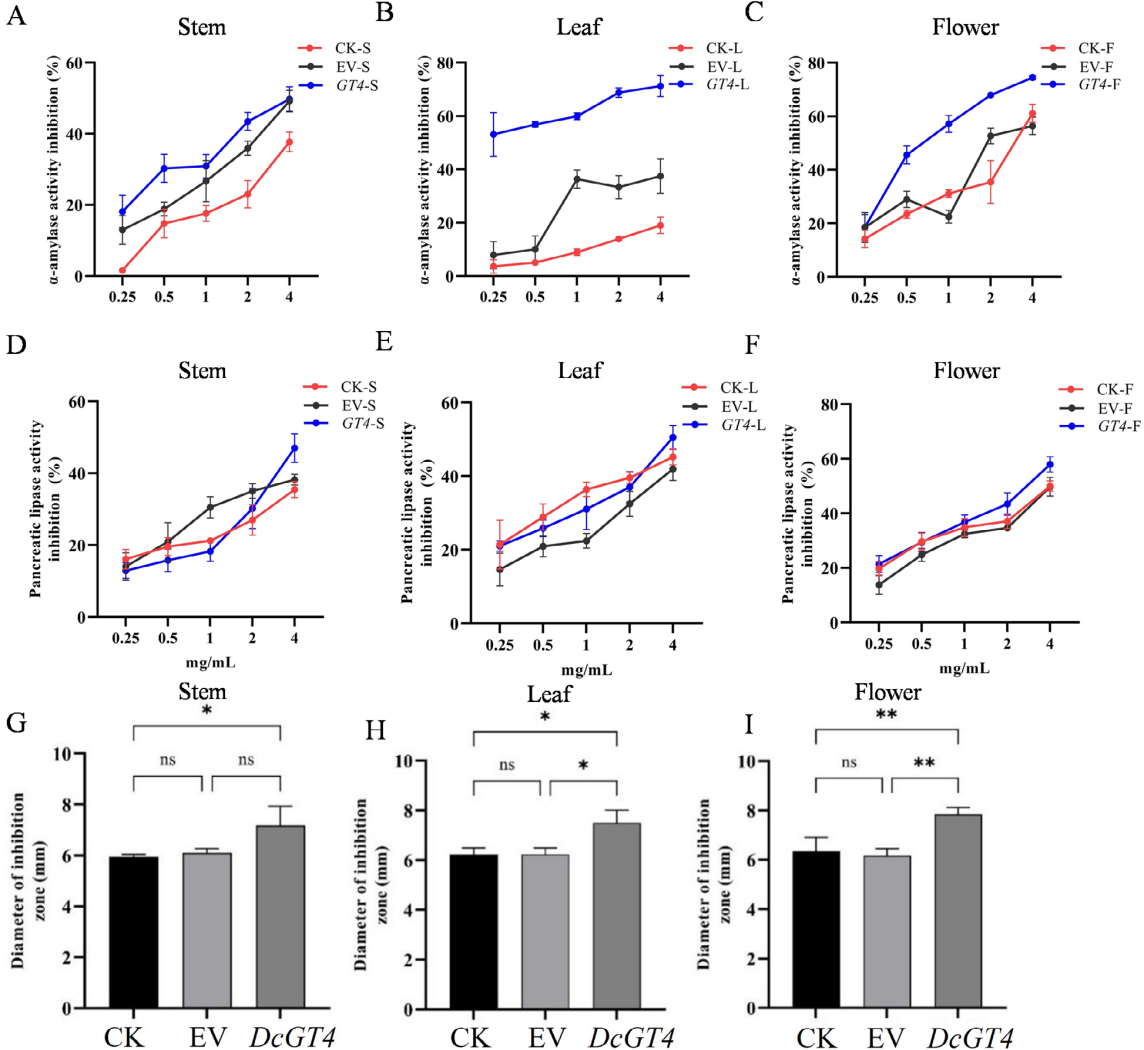
(A–C) α‐amylase inhibitory activities of different extracts of stem (A), leaf (B) and flower (C) in carnation, (D–F) Pancreatic lipase inhibitory activities of extracts from stem (D), leaf (E) and flower (F) in carnation, (G–I) Quantification of the inhibition zone for *Fusarium oxysporum f*. sp. *Dianthi* from stem (G), leaf (H) and flower (I). The results were expressed as mean values ± SD (*n* = 3). Different letters within the same figure indicate statistical difference (*p* < 0.05). (ns, *P* > 0.05; *, *P* < 0.05; **, *P* < 0.01).

The enzyme inhibition activities of extracts from WT, EV and *UGT73A93* carnation against α‐amylase and pancrelipase are shown in Figure 4. The inhibition rates of the *UGT73A93* carnation flower extract (4 mg/mL) against α‐amylase and pancrelipase were 74.44% and 57.93%, respectively (Figure 4C,F). The inhibition rates of the EV carnation flower extract (4 mg/mL) against α‐amylase and pancrelipase were 56.41% and 49.73%, respectively (Figure 4C,F). For the WT carnation flower extract (4 mg/mL), the inhibition rates against α‐amylase and pancrelipase were 61.12% and 50.01%, respectively (Figure 4C,F). Therefore, the inhibition rates of *UGT73A93* carnation flower extract against both α‐amylase and pancrelipase were higher than those of EV and WT carnation flower extracts. Additionally, the enzyme inhibition in the stems and leaves of *UGT73A93* carnation was higher than that in WT and EV (Figure 4A,B,D,E).

We also investigated the potential of *UGT73A93*, EV and WT extracts (at 1 mg/mL) to inhibit the growth of FOD. The results showed that *UGT73A93* extracts from stems, leaves and flowers exhibited higher activity against FOD growth compared to WT and EV (Figure 4G–I).

### 2.7 Proteins Binding to the *UGT73A93* Promoter

**FIGURE 5:**
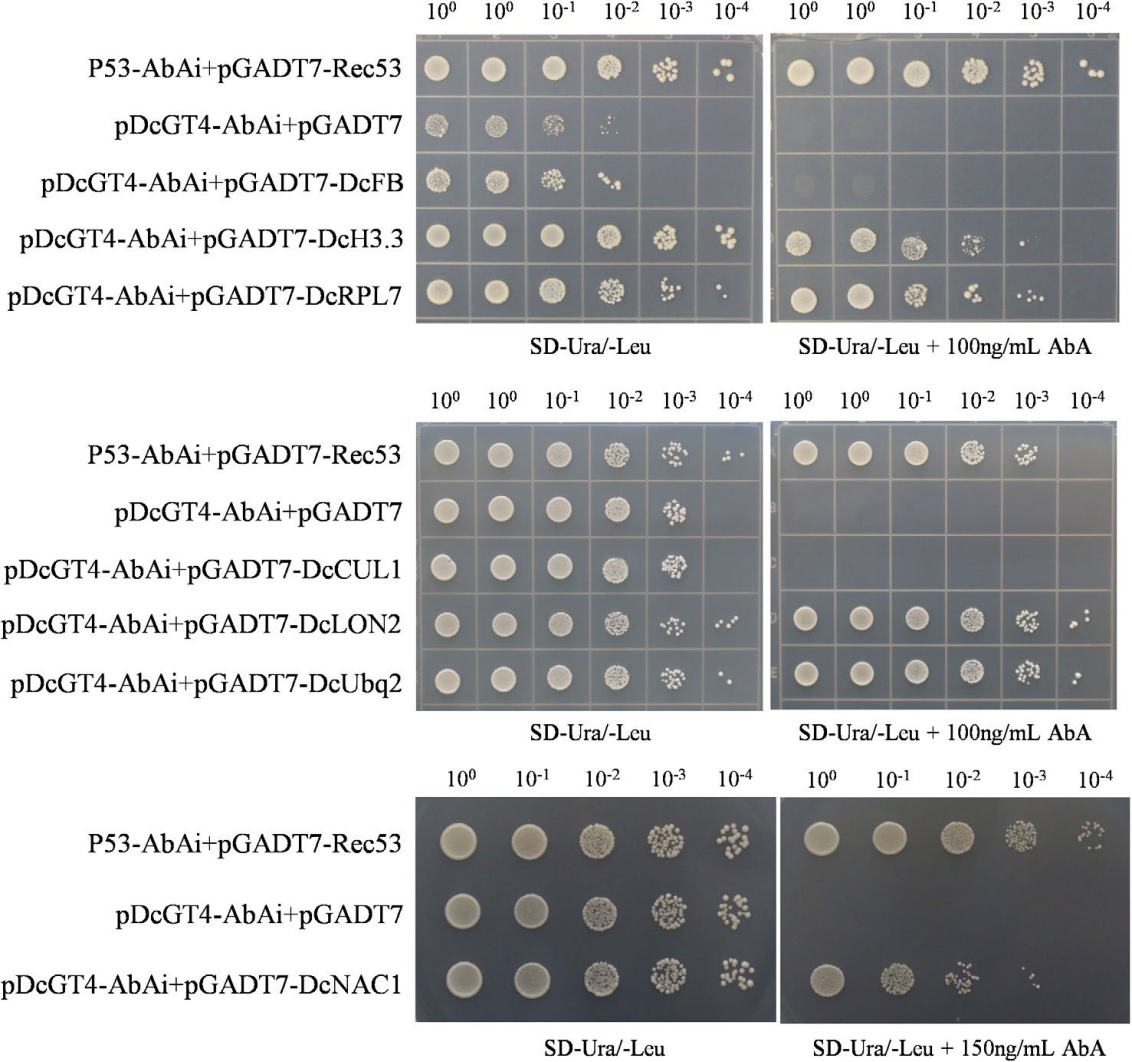
Y1H assay showing the interaction between DcFB, DcH3.3, DcRPL7, DcCUL1, DcLON2, DcUbq2, DcNAC1 and the *UGT73A93* (*DcGT4*) promoter. p53AbAi and pGADT7‐Rec53 were used as a positive control, pDcGT4‐AbAi and pGADT7 were used as a negative control. SD‐Ura/−Leu, yeast‐deficient medium (without Uracil and leucine); AbA, Aureobasidin A.

We constructed a yeast hybrid library for carnation, isolating a 1000 bp *UGT73A93* promoter sequence (Table S4). The *UGT73A93* promoter sequence was then cloned into the pAbAi vector to generate a decoy plasmid, which was used to screen for interacting proteins by pairing the decoy plasmid with the library. Seven interacting proteins were identified: Histone H3.3 (DcH3.3, GenBank accession number PV083450), Ribosomal protein L7 (DcRPL7, GenBank accession number PV083448), F‐box (DcFB, GenBank accession number PV083447), Cullin‐1 (DcCUL1, GenBank accession number PV083445), Lon protease 2 (DcLON2, GenBank accession number PV083449), DcUbq2 (GenBank accession number PV083446) and the DcNAC1 transcription factor (GenBank accession number PV083451). The Y1H one‐to‐one experiment was carried out, in which the p53‐AbAi receptor single‐transformed pGADT7‐Rec53 plasmid was used as a positive control on SD‐Ura/−Leu + AbA medium at the lowest inhibitory concentration. The pDcNAC1‐AbAi receptor was used as a negative control and did not grow on SD‐Ura/−Leu + AbA medium at the lowest inhibitory concentration. In the experimental group, pGADT7‐protein (DcH3.3, DcRPL7, DcFB, DcCUL1, DcLON2, DcUbq2 and DcNAC1) and pDcGT4‐AbAi vectors were co‐transformed into yeast. Yeast spots grew on the selective SD‐Ura/−Leu + AbA medium, indicating that five proteins (DcH3.3, DcRPL7, DcLON2, DcUbq2 and *DcNAC1*) likely interact with the promoter region of *UGT73A93*, while DcFB and DcCUL1 did not interact with the promoter region of *UGT73A93* (Figure 5).

**FIGURE 6:**
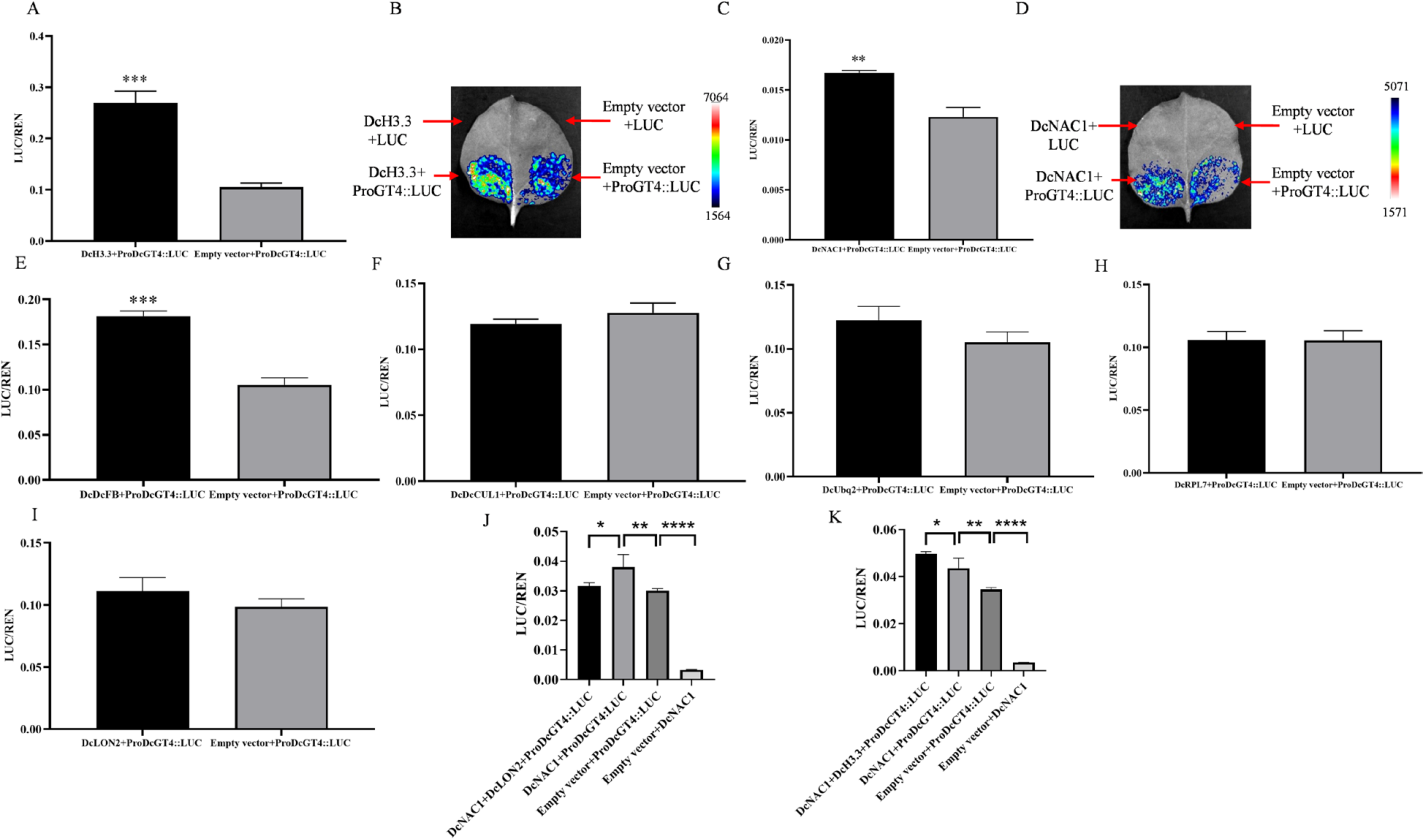
The luciferase report experiment in *N. benthamiana* protoplasts using luciferase (LUC)/Renilla luciferase (REN) assay (A, C, E–I) and *N. benthamiana* leaves (B, C) showed that between 7 proteins activated or not activated the *UGT73A93* (*DcGT4*) promoter. (J, K) The luciferase report experiment in tobacco showed that the addition of DcLON2 suppressed, while DcH3.3 enhanced the transcriptional activation of DcNAC1. Error bars represent ± SD (*n* = 3) from three replicates. Asterisks indicate significant differences by multiple *t*‐test (*p < 0.05, **p < 0.01, ***p < 0.001).

Next, dual‐luciferase assays were conducted to determine whether the proteins DcFB, DcH3.3, DcRPL7, DcCUL1, DcLON2, DcUbq2 and DcNAC1 could enhance the expression of *UGT73A93*. Protoplasts from *Arabidopsis thaliana* ( *A. thaliana* ) or leaves from *N. benthamiana* were co‐infiltrated with vectors containing 7 proteins, along with the promoters of *UGT73A93*. Compared to the empty vector (control), the expression of DcH3.3 enhanced the activity of the *UGT73A93* promoter. The activation of the reporter gene by DcH3.3 and DcNAC1 was 2.56‐fold and 1.34‐fold higher than the control, as determined by the luciferase (LUC)/Renilla luciferase (REN) assay (Figure 6A,C). Additionally, transcriptional activation of the *UGT73A93* promoter by DcH3.3 and DcNAC1 was confirmed in tobacco leaves (Figure 6B,D). These luciferase reporter experiments demonstrated that DcH3.3 and DcNAC1 significantly activated the *UGT73A93* promoter. Taken together, these findings indicate that DcH3.3 and DcNAC1 enhance flavonoid synthesis by directly binding to the *UGT73A93* promoter, thereby increasing gene expression.

The coexpression of DcFB with the promoters of *UGT73A93* significantly increased the expression of the reporter gene in dual‐luciferase assays (Figure 6E). However, DcFB did not interact with the promoter region of *UGT73A93* in the Y1H assay (Figure 5). The coexpression of DcRPL7, DcLON2, DcUbq2 and DcCUL1 with the promoters of UGT73A93 did not significantly affect the expression of the reporter gene in the dual‐luciferase assays (Figure 6F–I). Therefore, of the seven proteins tested, only DcH3.3 and DcNAC1 interact with the *UGT73A93* promoter and activate reporter gene expression.

To assess whether the protein complexes activate or repress transcription of the target gene *DcGT4*, we performed transient LUC complementation imaging assays using two combinations of recombinant plasmids: DcNAC1‐62‐SK with DcH3.3‐62‐SK and DcNAC1‐62‐SK with DcLON2‐62‐SK (Figure 6J,K). The *UGT73A93* (*DcGT4*) promoter was cloned into the pGreenII 0800‐LUC vector as the reporter. Compared with co‐expression of DcNAC1 alone with ProDcGT4::LUC, co‐expression of *DcNAC1* and *DcH3.3* resulted in a significantly higher LUC/REN ratio, indicating enhanced transcriptional activation. In contrast, co‐expression of *DcNAC1* and *DcLON2* yielded the opposite effect, with a markedly reduced LUC/REN ratio.

### 2.8 DcNAC1 Interacts With DcLON2

**FIGURE 7:**
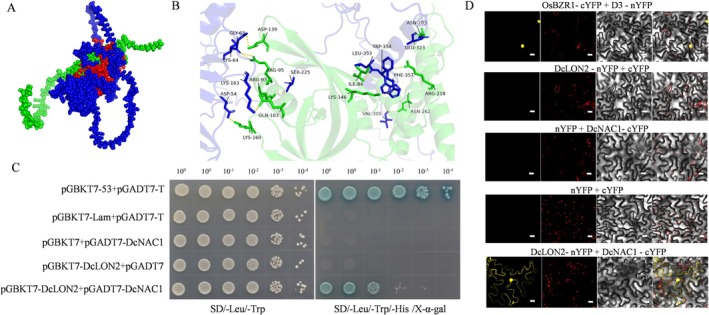
Interaction of the DcNAC1 and DcLON2 proteins. (A) AlphaFold 3 predicts structures of DcLON2 and DcNAC1 complexes. Red residues, predicted the interface of the DcLON2 and DcNAC1 proteins; blue, predicted DcNAC1 protein; green, predicted DcLON2 protein. (B) Structural model of interface DcNAC1‐DcLON2. (C) Interactions of the DcNAC1 and DcLON2 proteins revealed by yeast two‐hybrid experiments. (D) DcNAC1 interact DcLON2 in BiFC assays. Scale bars represent 20 mm.

We used AlphaFold 3 predictions to model protein–protein interactions. According to Homma et al. (2024), selecting results with ipTM ≥ 0.6 and pTM ≥ 0.5 scores yields good candidate complexes where both proteins have high‐confidence predicted folds. AlphaFold 3 predicted the binding of DcLON2 to DcNAC1 with slightly lower confidence (ipTM score of 0.53 and pTM score of 0.45) (Figure 7A,B). The red residues in Figure 7A are located at the interface between the DcLON2 and DcNAC1 proteins. The binding sites of the DcLON2 and DcNAC1 proteins are shown in Figure 7B. Additionally, yeast two‐hybrid experiments and bimolecular fluorescence complementation (BiFC) assays confirmed that DcLON2 can interact with DcNAC1 (Figure 7C,D).

## 3 Discussion

### 3.1 *UGT73A93* Participates in Flavonoid Accumulation

UDP‐glucose flavonoid 3′*‐O‐*glycosyltransferase (UF3GT) is a common enzyme that catalyses the glycosylation of both flavonol and anthocyanidin aglycons in vitro. Notably, in flavonoid biosynthesis, flavonoid 3′*‐O‐*glucosyltransferase plays an essential role as the initial step in glycosylation, before the synthesis of flavanone disaccharides or trisaccharides, which are the most abundant and characteristic FGs in carnation. In *Freesia hybrid*, Fh3GT2 preferentially glucosylates kaempferol, while Fh3GT1 controls the glucosylation of quercetin and anthocyanidins (Meng et al. 2019). Overexpression of *Fh3GT1* in *Petunia hybrida* results in a significant increase in cyanidin, peonidin and kaempferol content in transgenic flowers (Sun et al. 2017). Conversely, the silencing of the PeUFGT3 gene in *Phalaenopsis* leads to a decrease in anthocyanin content, resulting in faded flower colour (Chen et al. 2011). However, the diversity of function of UF3GT paralogous genes at both the biochemical and transcriptional levels remains largely unexplored. Both *Rd3GT1* and *Rd3GT6* catalyse the addition of UDP‐sugar to the 3‐OH position of anthocyanidins, with a preference for UDP‐Gal as the sugar donor and cyanidin as the most efficient substrate (Sun et al. 2022).

In our efforts to understand the flavonoid glycosylation system in carnation, the sequence of *UGT73A93* was cloned and its function analysed. When compared to the wild type and EV, transgenic tobacco plants expressing *UGT73A93* produced lighter pink flowers. Interestingly, the introduction of *UGT73A93* in tobacco led to an increase in flavonoid glycosides synthesis and a decrease in anthocyanin synthesis in the flowers, including elevated levels of kaempferol 3*‐O‐*rutinoside and kaempferol 3*‐O‐*sophoroside.

To further investigate the role of *UGT73A93* in flower colour development, antioxidant activity, enzyme inhibition and fungal inhibition, the gene was introduced into carnation. The transfer of *UGT73A93* into carnation restored flavonoid biosynthesis in the stems, leaves and flowers, confirming its functional role. Moreover, kaempferol derivatives, such as kaempferol‐3*‐O‐*sophorobiose‐7*‐O‐*glucoside, kaempferol 3*‐O‐*rutinoside and kaempferol 3*‐O‐*glucoside, were detected in the transgenic carnation. These observations suggest that *UGT73A93* can utilise kaempferol as a substrate and add UDP sugars to the 3‐OH position in both tobacco and carnation. The pale flower colour exhibited by *UGT73A93*‐overexpressing transgenic plants is mainly attributed to a redirection of flavonoid metabolic flux. UGT73A93 catalyses the glucosylation of flavonols and enhances the accumulation of kaempferol glycosides. This reinforces the flavonol branch and competitively diverts metabolic precursors from anthocyanin biosynthesis, leading to reduced anthocyanin accumulation and the resulting lightened flower coloration. Notably, transcript levels of *DcFLS* were downregulated in *UGT73A93*‐overexpressing lines, whereas flavonol glycoside contents increased markedly. This apparent discrepancy may imply post‐transcriptional regulation that stabilises the DcFLS protein or elevates its enzymatic activity. In future work, metabolomics will be applied to gain a more integrated understanding of the metabolic network modulated by UGT73A93.

Antioxidant assays indicated that *UGT73A93* in carnation extracts exhibited significantly higher DPPH, ABTS free radical scavenging ability and FRAP compared to the wild type and EV. Moreover, *UGT73A93* extracts exhibited strong inhibition of α‐amylase, pancreatic lipase and FOD. However, the kaempferol derivatives that accumulate in *DcGT4* transgenic lines, including kaempferol 3‐*O*‐rutinoside, kaempferol 3‐*O*‐sophoroside, kaempferol‐3‐*O*‐sophorobiose‐7‐O‐glucoside and kaempferol 3‐*O*‐glucoside, showed no antifungal activity against FOD (data not shown). We therefore speculate that other flavonoid metabolites present in the transgenic lines may contribute to the observed fungal resistance.

Therefore, the results obtained clearly demonstrate that *UGT73A93* is essential for flower colour development and can serve as a valuable molecular tool to improve flavonoid content and enhance anti‐fungal ability in plants. These findings could contribute to the exploitation of carnation as a natural resource for functional foods and nutraceutical ingredients. Although the present study functionally identified UGT73A93 as a flavonoid 3′*‐O‐*glucosyltransferase and revealed its physiological roles in flavonoid glycoside biosynthesis, detailed kinetic parameters including Km, Vmax and catalytic efficiency toward different flavonoid substrates were not determined in this work. Future in vitro enzymatic assays with purified protein will be necessary to precisely define its substrate specificity and catalytic efficiency, which will deepen our understanding of its biochemical function in planta.

### 3.2 DcNAC1 and DcH3.3 Co‐Activate the *UGT73A93* Promoter and Participate in Flavonoid Accumulation

The *NAC* gene family is one of the largest plant‐specific transcription factor families, with members playing key roles in various plant processes, including stress responses, secondary cell wall synthesis, lateral root development, yield potential, seed size, biomass production, ROS signalling, leaf senescence and programmed cell death (Nakashima et al. 2012; Olsen et al. 2005; Puranik et al. 2012; Singh et al. 2021; Srivastava et al. 2022). For example, MdNAC52 binds to the promoters of MdMYB9 and MdMYB11 to promote anthocyanin accumulation in apple (Sun et al. 2019). Overexpression of *MdNAC1* in apple fruits and calli significantly increased anthocyanin content, with *MdNAC1* interacting with *MdbZIP23* to activate the transcription of *MdUFGT* (Liu, Mei, et al. 2023). Similarly, PpNAC1 and PpMYB10.1 synergistically activate the expression of *PpUFGT*, which is involved in anthocyanin biosynthesis in peach tree leaves (Meng et al. 2023). In *Arabidopsis*, the ANAC078 protein is associated with the induction of flavonoid biosynthesis genes, leading to anthocyanin accumulation in response to high‐light stress.

H3.3, a variant of the H3 histone, is found in various eukaryotes and is typically associated with transcription initiation and/or elongation activities of RNA Polymerase II in animals (Goldberg et al. 2010; Mito et al. 2005). Tafessu et al. (2023) demonstrated that H3.3 promotes accessibility, transcription factor binding and the recruitment of transcriptional coactivators, such as p300 and BRD4, to active promoters in embryonic stem cells through genomic analyses. Shu et al. (2014) found that H3.3 is present on active promoters of strongly regulated genes, suggesting a role in transcriptional regulation. Furthermore, Liu, Yin, et al. (2023) proposed that the ASF1A/1B‐HIRA complex mediates H3.3 deposition at its direct targets to active transcription, contributing to the regulatory networks governing male gamete development in *Arabidopsis*. Zhao et al. (2022) further indicated that the enrichment of H3.3 at the 5′ region may facilitate chromatin opening, promoting transcription factor binding in *Arabidopsis* seeds and during germination. Our findings found that both DcNAC1 and DcH3.3 bind to the promoters of UGT73A93 (DcGT4), activating the transcription of this gene.

### 3.3 AlphaFold 3 Predicted the Interaction Between DcLON2 and DcNAC1

The protease Lon plays a critical role in the degradation of misfolded proteins, particularly under oxidative stress conditions (Voos and Pollecker 2020). LON2 is involved in matrix protein degradation during peroxisome content remodelling, providing evidence for the existence of pexophagy in plants. It also indicates that peroxisomal destruction via autophagy is enhanced when LON2 is absent (Farmer et al. 2013). However, the interaction between LON2 and NAC1 has not been previously reported.

AlphaFold 3 is a core tool for analysing protein complex structures. Its high prediction accuracy (Abramson et al. 2024) and intuitiveness (Zhao et al. 2025) have unlocked new applications in various fields, specifically in plant science, including improving crop breeding and predicting the structures of plant‐specific proteins involved in stress responses and signalling pathways. Wang et al. (2025) predicted the DNA recognition mechanism of OsDREB1A using AlphaFold 3 and then obtained the above results through validation (Wang et al. 2025). Kim et al. (2025) exploring the structural diversity and evolution of the D1 Subunit of Photosystem II Using AlphaFold 3 (Kim et al. 2025). Guided by AlphaFold 3‐based AI predictions, we identified a putative interaction between DcNAC1 and DcLON2 and designed targeted experiments to test this hypothesis. The results provide evidence for a physical association between the two proteins, but do not establish a directional regulatory relationship. To address this, we performed dual‐luciferase assays, which demonstrated that DcLON2 and DcNAC1 bind to the promoter of *UGT73A93* (*DcGT4*) and downregulate the transcription of this gene.

**FIGURE 8:**
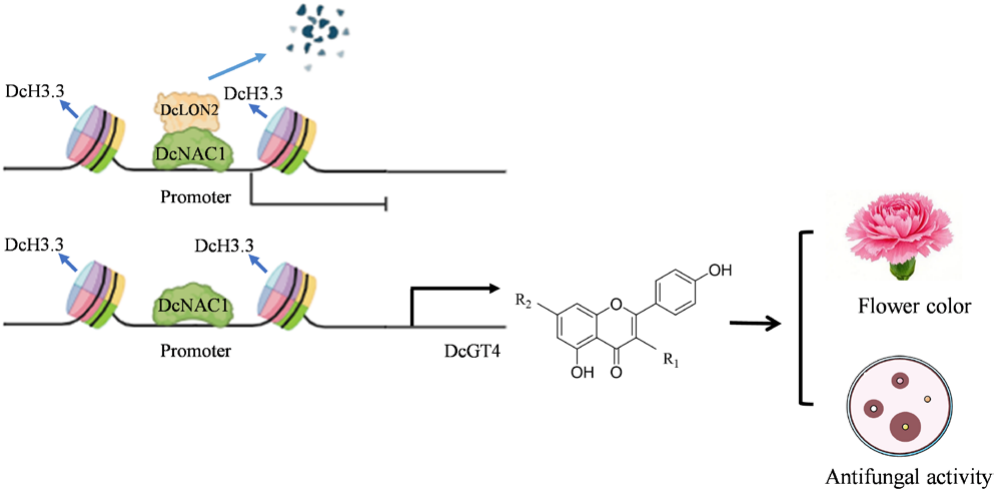
A regulatory model explaining flavonoid biosynthesis in carnation. *DcH3.3* is loaded at the promoters and establishes chromatin accessibility, which was largely maintained probably by the binding of DcNAC1 transcription factors and this may allow UGT73A93 (DcGT4) transcriptional regulation during plant development. DcLON2 was responsible for the degradation of DcNAC1 proteins and prevented gene transcription.

Our findings reveal that both DcNAC1 and DcH3.3 bind to the promoters of *UGT73A93* (*DcGT4*), activating the transcription of this gene. While DcH3.3 may function independently of DcNAC1, it is not a transcription factor in carnation. Instead, it contributes to chromatin opening, facilitating the binding of DcNAC1 to the promoter. Collectively, these results show that the DcNAC1 transcription factor regulates flavonoid accumulation by directly binding to the *UGT73A93* promoter. DcLON2 interacts with DcNAC1 and downregulates the transcription of *UGT73A93* by mediating the degradation of DcNAC1 (Figure 8).

## 4 Conclusions

This study identified DcNAC1 as a key regulator that targeted the flavonoid activator UGT73A93, thereby enhancing flavonoid biosynthesis in carnation. Moreover, DcNAC1 and DcH3.3 worked synergistically to activate *UGT73A93* expression, further promoting flavonoid biosynthesis. Therefore, we revealed a novel flavonoid synthesis mechanism in carnation. The genes responsible for the biosynthesis of diverse flavonoid glycosides in carnation could serve as a gene bank for microbial engineering. Carnation could also serve as a chassis for nutritional food production by introducing unique metabolic pathways.

## 5 Materials and Methods

### 5.1 Chemicals and Materials

Kaempferol‐3*‐O‐*sophorobiose‐7*‐O‐*glucoside, kaempferol 3*‐O‐*sophoroside, kaempferol 3*‐O‐*rutinoside and kaempferol 3*‐O‐*glucoside (purity > 98%) were extracted in our previous study and the compounds were characterised using NMR and MS techniques (Wang et al. 2024).

Carnations and tobacco were obtained from Yunnan University of Chinese Medicine in Kunming, China. Buds at three developmental stages, along with petals, calyx, leaves and stems, were separated, immediately frozen in liquid nitrogen and stored at −80°C.

*Escherichia coli* ATCC 25922, *Staphylococcus aureus* ATCC 6538 and *Pseudomonas aeruginosa* ATCC 9027 were purchased from Haibo Biotechnology Co. Ltd. (Qingdao, China). *Fusarium oxysporum* f. sp. *dianthi* was obtained from the Yunnan Academy of Agricultural Sciences, China. Mueller–Hinton (MH) broth, MH agar and potato dextrose agar (PDA) media were purchased from Guangdong Huankai Microbiology Technology Co. Ltd. (China).

### 5.2 RNA Extraction, cDNA Synthesis, Gene Cloning and RT‐qPCR Analysis

The frozen samples were ground using a porcelain mortar before RNA extraction. RNA extraction was performed according to the instructions of the Plant Total RNA Extraction Kit (Invitrogen, USA). Total RNA (1 μg) was used for reverse transcription with the Reverse Transcription Kit (Yeasen, China). The primers for cloning the *UGT73A93* gene and its promoter are shown in Table S1. The associated UGT amino acid sequences were obtained from NCBI under the accession numbers listed in Table S2. A phylogenetic tree was constructed using the neighbour‐joining method in MEGA 11. RT‐qPCR was performed using Hieff qPCR SYBR Green Master Mix (Yeasen, China) on the LC96 (Roche, Switzerland). The GAPDH was used as the reference gene (Zhou et al. 2022). Gene expression was calculated using the 2^−ΔΔCt^ method. The primers used are shown in Table S1 and are based on the carnation genome (http://carnation.kazusa.or.jp/index.html) database gene sequences (Yagi et al. 2014). Each reaction was conducted in triplicate and the results were analysed using LightCycler 96 Software (Roche, Switzerland).

### 5.3 Extraction, Identification and Quantification of Flavonol Glycosides

Extraction of flavonol glycosides (FGs) was performed as described in a previous study with minor modifications (Wang et al. 2022). Flesh carnation and tobacco flowers, stems and leaf samples were ground into powder under liquid nitrogen. Approximately 2.0 g of carnation powder was sonicated in 30 mL of methanol solution and 2.5 g of tobacco powder was sonicated in 15 mL of methanol solution. The methanol solution, which consisted of 90% methanol and 10% glacial acetic acid, was sonicated for 48 h at room temperature to extract kaempferol‐3*‐O‐*sophorobiose‐7*‐O‐*glucoside, kaempferol 3*‐O‐*rutinoside and kaempferol 3*‐O‐*glucoside from carnation and kaempferol 3*‐O‐*sophoroside and kaempferol 3*‐O‐*rutinoside from tobacco. After centrifugation at 10 000 × *g* for 10 min at 4°C, the supernatant was collected and filtered through a 0.22 μm micropore membrane filter for HPLC analysis.

FGs were determined according to Wang et al. (2022) with slight modifications. Flavonoids were separated using an Agilent 1260 Infinity LC system (Agilent, Japan) and an Agilent ZORBAX SB‐C18 column. The specific chromatographic conditions for tobacco extraction were as follows: mobile phase A consisted of 1.5% phosphoric acid, 40% acetonitrile and 50% glacial acetic acid, while mobile phase B consisted of 1.5% phosphoric acid dissolved in pure water, with gradient elution from 10% to 40% in channel A. The chromatographic conditions for carnation extraction were similar, with gradient elution from 10% to 30% in channel A. Quantification of FGs was performed using calibration curves created with corresponding commercial standards (Supporting Information Table S3).

### 5.4 Obtaining and Validating the *UGT73A93* Transgenic Carnation and Tobacco Lines

Primers were used to amplify the *UGT73A93* genes (Table S1), which were then digested using the restriction endonucleases AarI. The pBWA(V)HS vector was digested with BsaI/Eco31I and the target gene was subsequently ligated into the vector. Next, 5 μL of the ligation product was used to transform chemically competent DH5α cells. The *UGT73A93* gene PCR primers were used to screen single colonies with the expected bands (Supporting Information Table S1). Hygromycin primers were used to screen transgenic carnation and tobacco plants (Supporting Information Table S1).

The pBWA(V)HS‐UGT73A93 recombinant vector and the empty vector (EV, as control) were transferred into *Agrobacterium tumefaciens* strain GV3101. Transgenic tobacco was generated by transforming leaf discs with *A. tumefaciens* , as previously described (Park et al. 2019). The explants were cultured and selected on shoot‐inducing media containing 25 mg/L hygromycin. The regenerated shoots were subsequently transplanted to soil and grown in the greenhouse. The transgenic tobacco plants were grown to maturity and seeds were obtained after self‐pollination. Representative T1 and T3 transgenic lines were selected for further analysis based on observed changes in petal colour. Transgenic carnations were generated using *A. tumefaciens* as described previously (Zhou et al. 2022). The regenerated plantlets were acclimatised and grown in the greenhouse and tissue from the transgenic carnation plants was collected for further analysis.

### 5.5 Measurement of TAC and TFC in *UGT73A93* Transgenic Lines, EV and CK in Carnation and Tobacco

Carnation and tobacco flowers, stems and leaves from EV, CK and *UGT73A93* transgenic lines were freeze‐dried to a constant weight and ground into powders. The measurement and extraction of total flavonoid content (TFC) were performed according to the method described by Wang et al. (2024) with slight modifications. Metabolites were extracted from 100 mg of tobacco powder using 9 mL of 50% (v/v) ethanol and from 100 mg of carnation powder using 2.5 mL of 70% (v/v) ethanol. The tobacco samples were sonicated three times at 75°C for 60 min each (100 W), while the carnation samples were sonicated three times at 60°C for 45 min each (100 W). Both mixtures were then centrifuged at 10 000 × *g* for 10 min. Chloroform was added to the supernatants to remove chlorophyll, followed by another centrifugation at 10 000 × *g* for 10 min. The resulting supernatants were transferred to Eppendorf tubes and stored at −70°C in the dark for subsequent analysis. All analyses were performed in triplicate for each plant sample. The flavonoid content, reported as milligrams of rutin per gram of sample (mg/g), was determined using rutin as the reference standard.

Total anthocyanin content (TAC) measurements and extraction were performed according to the method of Li (2022) with slight modifications. The metabolites were extracted from 100 mg of powder using 2.5 mL of 50% ethanol (with 0.8% hydrochloric acid) solution. The mixtures were sonicated for 45 min (60°C, 100 W) three times and then centrifuged at 8000 g for 10 min. The supernatants were transferred to Eppendorf tubes and stored in the dark at −70°C for subsequent analysis. Cyanidin‐3*‐O‐*glucoside was used as the standard and the results were expressed as milligrams of cyanidin‐3*‐O‐*glucoside per gram of sample (mg/g).

### 5.6 Antioxidant Assays in UGT73A93, EV and CK Carnation and Tobacco

The antioxidant capacity of plant samples was determined using the 2,2‐diphenyl‐1‐picrylhydrazyl (DPPH) assay, the 2, 2‐azino‐bis‐3‐ethylbenzothiazoline‐6‐sulfonic acid (ABTS) assay and the ferric‐reducing antioxidant power (FRAP) assay, following the method described by Wang et al. (2024).

### 5.7 Valuation of Petal Colour

The lightness (L*) and chromatic components a* and b* of the Commission Internationale de l'Eclairage (CIE) L*a*b* colour space of each petal were measured using a colorimeter (3nh, China) on three flower petals. The colorimetric values were measured in triplicate.

### 5.8 Determination of Inhibition of α‐Amylase and Pancreatic Lipase and Tyrosinase in *UGT73A93* , EV and CK Carnation and Tobacco

The inhibition of α‐amylase and pancreatic lipase was measured according to the methods of Zhang et al. (2017) and Yuan et al. (2018).

### 5.9 Antifungal Activity of *UGT73A93* , EV and CK Carnation and Tobacco

The antimicrobial activity of plant extracts was evaluated using the disk diffusion method (Klančnik et al. 2010). *Fusarium oxysporum* f. sp. *dianthi* was cultured in Mueller–Hinton broth medium at 28°C for 48 h. The plant extracts were applied to filter paper discs, maintaining a final concentration of 1 mg/mL. Mueller–Hilton agar was poured into Petri dishes and each dish was inoculated with 800 μL of microbial suspension (1.5 × 10^6^ CFU/mL), followed by the placement of filter paper discs containing plant extracts from different plant parts. A filter paper disc containing 5 mg of tetracycline hydrochloride (Sigma, USA) was included as a positive control and a disc containing only the solvent (without plant extract) was used as a negative control.

### 5.10 Yeast One‐Hybrid Assays

The Matchmaker Gold Yeast One‐Hybrid Library Screening System (Clontech, USA) was used for yeast assays. The Y1HGold yeast strain was transformed with pAbAi vectors containing the baits. The bait, cloned from the target promoters of *UGT73A93* (*DcGT4*), was designated as pDcGT4‐AbAi. A yeast library was constructed by cloning cDNA libraries of mRNAs from carnation buds and flowers into the pGADT7 vector. Yeast library screening was performed using pGT4‐AbAi according to the manufacturer's manual (Clontech, USA). The primers used are shown in Table S1. The recombinant plasmid containing pDcGT4‐AbAi was used as the bait for screening the cDNA library.

The coding sequences (CDS) of Histone H3.3 (DcH3.3), Ribosomal protein L7 (DcRPL7), F‐box (DcFB), Cullin‐1 (DcCUL1), Lon protease 2 (DcLON2), DcUbq2 and DcNAC1 from carnation were fused to the pGADT7 vector. The recombinant pGADT7 vectors were then transformed into the Y1HGold yeast strain, along with the reporter linearised plasmid pDcGT4‐AbAi, to determine protein–DNA interactions. Positively transformed yeast cells were identified by spotting serial dilutions (1:1 to 1:10 000) onto SD/−Ura/−Leu medium supplemented with 100 or 150 ng/mL Aureobasidin A (AbA).

### 5.11 Dual‐Luciferase Assay

Dual‐luciferase assays were performed as described in a previous study (Zhong et al. 2020). The promoter region of *UGT73A93* (*DcGT4*) was cloned into the pGreenII 0800‐LUC vector. The assays were carried out in *Arabidopsis thaliana* protoplasts using the Dual Luciferase Reporter Assay Kit (Vazyme, China). The *REN* gene (Renilla luciferase) in the vector served as an internal control. The ratio of LUC to REN was used to represent the activity of the *UGT73A93* (*DcGT4*) promoter, with or without the effect of DcH3.3, DcLON2 and DcNAC1.

The recombinant plasmids pGreenII 0800‐LUC‐DcGT4pro, pGreenII‐62‐SK‐DcNAC1 and pGreenII‐62‐SK‐DcH3.3 were constructed and transformed into *A. tumefaciens* strain GV3103. The two GV3101 strains, each containing one of the recombinant plasmids, were mixed in equal proportions and co‐injected into *N. benthamiana* leaves. After 48 h of incubation in darkness, fluorescence was detected using the Tanon Chemi Doc 5200 T system (Tanon, China).

### 5.12 Yeast Two‐Hybrid Assays

The entire CDS of *DcNAC* was cloned and inserted into the EcoRI and BamHI sites of the pGADT7 vector. Similarly, the complete CDS of *DcLON2* was cloned and inserted between the EcoRI and BamHI sites of the pGBKT7‐ccdb vector. Y2H analysis was performed following the protocol described by Zhong et al. (2020).

### 5.13 Bimolecular Fluorescence Complementation Assays

For the BiFC assay, the full‐length coding sequences of the *DcLON2* genes were fused with N‐terminal YFP and those of *DcNAC1* were fused with C‐terminal YFP. OsBZR1‐cYFP + D3‐nYFP was used as the positive control, while DcLON2‐nYFP + cYFP, nYFP + DcNAC1‐cYFP and nYFP + cYFP served as negative controls.

### 5.14 Statistical Analysis

Statistical analyses were conducted using one‐way analysis of variance (ANOVA) or Student's *t‐*test (*p < 0.05; **p < 0.01; ***p < 0.001). All data were obtained from at least three biological replicates and error bars indicate standard deviation (SD). Figures were produced using GraphPad Prism 8.0 (GraphPad Software Inc., USA) and OriginPro 2021 (OriginLab Corporation, USA).

## Author Contributions

X.Z. conceived and designed the study. Q.X., X.Y., Y.Z., S.Z. and Y.T. performed experiments. X.Y. provided assistance. X.Z. and Q.X. analysed the data and wrote the manuscript. Y.Z., revised the manuscript. All authors read and approved the final manuscript.

## Funding

This work was supported by National Natural Science Foundation of China (32560746), Yunnan Provincial Science and Technology Department‐Applied Basic Research Joint Special Funds of Chinese Medicine (202001AZ070001‐012).

## Conflicts of Interest

The authors declare no conflicts of interest.

## Supporting information


**Figure S1:** Functional characterization of *UGT73A93* (*DcGT4*) gene following its overexpression in T3 generation transgenic tobacco lines (T1). (A) Differences in colour between wild‐type (WT), empty vector (EV) and transgenic tobacco flowers. (B) The values of L*, a* and b* classified by the CIELAB systems. (C–E) Expression of *UGT73A93* normalised to *NbGAPDH* gene in WT, EV and transgenic lines of stem (C), leaf (D) and flower (E). (F‐H) Contents of the total flavonoids in stem (F), leaf (G) and flower (H). (I) Contents of the total anthocyanins in flower. (J) HPLC chromatograms of flavonols glucosylated in vitro. (K, L) Contents of Kaempferol 3‐*O*‐sophoroside and Kaempferol 3‐*O*‐rutinoside in flower. Error bars represent ± SD from three replicates. Asterisks indicate significant differences by multiple *t*‐test (ns, *p* > 0.05; *p < 0.05; **p < 0.01; ***p < 0.001; ****p < 0.0001). Scale bars represent 1 cm.
**Figure S2:** Ferric ion reducing antioxidant power (FRAP) of stem (A), leaf (B) and flower (C) extraction in T1 generation transgenic tobacco.
**Figure S3:** Ferric ion reducing antioxidant power (FRAP) of stem (A), leaf (B) and flower (C) extraction in T3 generation transgenic tobacco.
**Figure S4:** Expression profiles of flavonoid‐related biosynthetic genes in flowers of transgenic carnation lines carrying *UGT73A93* (*DcGT4*) genes. The expression levels of *DcFLS* (A), *DcGT5* (B), *DcF3′H* (C), *DcDFR* (D) gene in carnation stems, leave and flower. FLS, flavonol synthase; F3′H, flavonoid 3′‐hydroxylase; DFR, dihydroffavonol 4‐reductase; GT5, UDP‐glycose flavonoid glycosyltransferase 5. Error bars in represent ± SEM from three replicates. Asterisks indicate significant differences by multiple *t*‐test (ns, *p* > 0.05; *p < 0.05; **p < 0.01; ***p < 0.001; ****p < 0.0001).
**Figure S5:** Ferric ion reducing antioxidant power (FRAP) of stem (A), leaf (B) and flower (C) extraction in carnation.

**Table S1:** Primers used in this study. The underlines represent the homologous arms or restriction sites.
**Table S2:** Sequence accession numbers used in the phylogenetic.
**Table S3:** Calibration curves of four flavonol glycosides.
**Table S4:** The promoter sequence data for dual‐LUC transient expression assay.
**Table S5:** Antioxidant activity of total flavonoids in different parts of T3 generation transgenic tobacco.

## Data Availability

The data that support the findings of this study are available in the Supporting Informations S1 and S2 of this article.

## References

[pbi70674-bib-0001] Abramson, J. , J. Adler , J. Dunger , et al. 2024. “Accurate Structure Prediction of Biomolecular Interactions With Alphafold 3.” Nature 630: 493–500. 38718835 10.1038/s41586-024-07487-w PMC11168924

[pbi70674-bib-0002] Alseekh, S. , L. P. de Souza , M. Benina , and A. R. Fernie . 2020. “The Style and Substance of Plant Flavonoid Decoration; Towards Defining Both Structure and Function.” Phytochemistry 174: 112347. 32203741 10.1016/j.phytochem.2020.112347

[pbi70674-bib-0003] Ardila, H. D. , S. T. Martínez , and B. L. Higuera . 2013. “Levels of Constitutive Flavonoid Biosynthetic Enzymes in Carnation ( *Dianthus caryophyllus* L.) Cultivars With Differential Response to Fusarium Oxysporum F. Sp. Dianthi.” Acta Physiologiae Plantarum 35: 1233–1245.

[pbi70674-bib-0004] Bondonno, N. P. , F. Dalgaard , K. Murray , et al. 2021. “Higher Habitual Flavonoid Intakes Are Associated With a Lower Incidence of Diabetes.” Journal of Nutrition 151: 3533–3542. 34313759 10.1093/jn/nxab269 PMC8562076

[pbi70674-bib-0005] Chen, W.‐H. , C.‐Y. Hsu , H.‐Y. Cheng , H. Chang , H. H. Chen , and M. J. Ger . 2011. “Downregulation of Putative Udp‐Glucose: Flavonoid 3‐O‐Glucosyltransferase Gene Alters Fower Coloring in Phalaenopsis.” Plant Cell Reports 30: 1007–1017. 21274540 10.1007/s00299-011-1006-1

[pbi70674-bib-0006] Farmer, L. M. , M. A. Rinaldi , P. G. Young , C. H. Danan , S. E. Burkhart , and B. Bartel . 2013. “Disrupting Autophagy Restores Peroxisome Function to an Arabidopsis Lon2 Mutant and Reveals a Role for the Lon2 Protease in Peroxisomal Matrix Protein Degradation.” Plant Cell 25: 4085–4100. 24179123 10.1105/tpc.113.113407 PMC3877801

[pbi70674-bib-0007] Filgueira‐Duarte, J. J. , W. A. Gómez‐Corredor , and D. Londoño‐Serna . 2024. “The Resistance of Carnation ( *Dianthus caryophyllus* L.) to Fusarium Oxysporum F.Sp. Dianthi Is a Multigene‐Multivariate Phenomenon.” Tropical Plant Pathology 49: 489–501.

[pbi70674-bib-0008] Goldberg, A. D. , L. A. Banaszynski , K.‐M. Noh , et al. 2010. “Distinct Factors Control Histone Variant H3. 3 Localization at Specific Genomic Regions.” Cell 140: 678–691. 20211137 10.1016/j.cell.2010.01.003 PMC2885838

[pbi70674-bib-0009] Homma, F. , J. Lyu , and R. A. van der Hoorn . 2024. “Using Alphafold Multimer to Discover Interkingdom Protein–Protein Interactions.” Plant Journal 120: 19–28. 10.1111/tpj.16969 39152709

[pbi70674-bib-0010] Kim, T. D. , D. Pretorius , J. W. Murray , and T. Cardona . 2025. “Exploring the Structural Diversity and Evolution of the D1 Subunit of Photosystem ii Using Alphafold and Foldtree.” Physiologia Plantarum 177: e70284. 40401773 10.1111/ppl.70284 PMC12096807

[pbi70674-bib-0011] Klančnik, A. , S. Piskernik , B. Jeršek , and S. S. Možina . 2010. “Evaluation of Diffusion and Dilution Methods to Determine the Antibacterial Activity of Plant Extracts.” Journal of Microbiological Methods 81: 121–126. 20171250 10.1016/j.mimet.2010.02.004

[pbi70674-bib-0012] Li, T. , Y. Zhao , L. Yuan , et al. 2024. “Total Dietary Favonoid Intake and Risk of Cardiometabolic Diseases: A Dose‐Response Meta‐Analysis of Prospective Cohort Studies.” Critical Reviews in Food Science and Nutrition 64: 2760–2772. 36148848 10.1080/10408398.2022.2126427

[pbi70674-bib-0013] Li, X. 2022. “Determination and Analysis of Anthocyanin Content in Grape.” Xiandai Shipin 28: 179–182.

[pbi70674-bib-0014] Liu, K. , C. Yin , W. Ye , et al. 2023. “Histone Variant H3.3 Controls Arabidopsis Fertility by Regulating Male Gamete Development.” Plant and Cell Physiology 65: 68–78. 10.1093/pcp/pcad119 37814936

[pbi70674-bib-0015] Liu, W. , Z. Mei , L. Yu , et al. 2023. “The Aba‐Induced Nac Transcription Factor Mdnac1 Interacts With a Bzip‐Type Transcription Factor to Promote Anthocyanin Synthesis in Red‐Fleshed Apples.” Horticulture Research 10: uhad049. 37200839 10.1093/hr/uhad049 PMC10186271

[pbi70674-bib-0016] Meng, J. , S. Sun , A. Li , et al. 2023. “A Nac Transcription Factor, Ppnac1, Regulates the Expression of Ppmyb10. 1 to Promote Anthocyanin Biosynthesis in the Leaves of Peach Trees in Autumn.” Horticulture Advances 1: 8.

[pbi70674-bib-0017] Meng, X. , Y. Li , T. Zhou , et al. 2019. “Functional Differentiation of Duplicated Flavonoid 3‐O‐Glycosyltransferases in the Flavonol and Anthocyanin Biosynthesis of Freesia Hybrida.” Frontiers in Plant Science 10: 1330. 31681396 10.3389/fpls.2019.01330 PMC6813240

[pbi70674-bib-0018] Mito, Y. , J. G. Henikoff , and S. Henikoff . 2005. “Genome‐Scale Profiling of Histone H3.3 Replacement Patterns.” Nature Genetics 37: 1090–1097. 16155569 10.1038/ng1637

[pbi70674-bib-0019] Morimoto, H. , Y. Ando , H. Sugihara , T. Narumi‐Kawasaki , T. Takamura , and S. Fukai . 2021. “Information on Flower Coloration and Pigmentation in Current Carnation Cultivars for Use in Future Flower‐Color Breeding.” Horticulture Journal 90: 428–449.

[pbi70674-bib-0020] Nakashima, K. , H. Takasaki , J. Mizoi , K. Shinozaki , and K. Yamaguchi‐Shinozaki . 2012. “Nac Transcription Factors in Plant Abiotic Stress Responses.” Biochimica et Biophysica Acta (BBA) ‐ Gene Regulatory Mechanisms 1819: 97–103. 22037288 10.1016/j.bbagrm.2011.10.005

[pbi70674-bib-0021] Nakayama, M. 2020. “Flower Pigments Responsible for Cyanic, Yellow, and Cream‐White Coloration in Carnation.” In The Carnation Genome, 61–79. Springer Singapore.

[pbi70674-bib-0022] Ogata, J. , Y. Itoh , M. Ishida , H. Yoshida , and Y. Ozeki . 2004. “Cloning and Heterologous Expression of Cdnas Encoding Flavonoid Glucosyltransferases From *Dianthus caryophyllus* .” Plant Biotechnology 21: 367–375.

[pbi70674-bib-0023] Olsen, A. N. , H. A. Ernst , L. L. Leggio , and K. Skriver . 2005. “Nac Transcription Factors: Structurally Distinct, Functionally Diverse.” Trends in Plant Science 10: 79–87. 15708345 10.1016/j.tplants.2004.12.010

[pbi70674-bib-0024] Park, S. , D.‐H. Kim , B.‐R. Park , J.‐Y. Lee , and S.‐H. Lim . 2019. “Molecular and Functional Characterization of *Oryza sativa* Flavonol Synthase (Osfls), a Bifunctional Dioxygenase.” Journal of Agricultural and Food Chemistry 67: 7399–7409. 31244203 10.1021/acs.jafc.9b02142

[pbi70674-bib-0025] Pérez Mora, W. , L. M. Melgarejo , and H. D. Ardila . 2021. “Effectiveness of Some Resistance Inducers for Controlling Carnation Vascular Wilting Caused by Fusarium Oxysporum F. Sp. Dianthi.” Archives of Phytopathology and Plant Protection 54: 886–902.

[pbi70674-bib-0026] Puranik, S. , P. P. Sahu , P. S. Srivastava , and M. Prasad . 2012. “Nac Proteins: Regulation and Role in Stress Tolerance.” Trends in Plant Science 17: 369–381. 22445067 10.1016/j.tplants.2012.02.004

[pbi70674-bib-0027] Ren, C. , J. Qian , Y. Wang , et al. 2025. “Flavonoid Udp‐Glycosyltransferase in Plants: Functional Identification, Substrate Recognition Mechanism, and Biotechnology Application.” Phytochemistry Reviews 24: 4451–4474.

[pbi70674-bib-0028] Sharma, P. , A. K. Nath , A. Kumar , and A. Shyam . 2024. “Genetic Improvement of Carnation.” In Genetic Engineering of Crop Plants for Food and Health Security: Volume 1, 57–68. Springer.

[pbi70674-bib-0029] Shu, H. , M. Nakamura , A. Siretskiy , et al. 2014. “Arabidopsis Replacement Histone Variant H3.3 Occupies Promoters of Regulated Genes.” Genome Biology 15: R62. 24708891 10.1186/gb-2014-15-4-r62 PMC4054674

[pbi70674-bib-0030] Singh, S. , H. Koyama , K. K. Bhati , and A. Alok . 2021. “The Biotechnological Importance of the Plant‐Specific Nac Transcription Factor Family in Crop Improvement.” Journal of Plant Research 134: 475–495. 33616799 10.1007/s10265-021-01270-y PMC8106581

[pbi70674-bib-0031] Srivastava, R. , Y. Kobayashi , H. Koyama , and L. Sahoo . 2022. “Overexpression of Cowpea Nac Transcription Factors Promoted Growth and Stress Tolerance by Boosting Photosynthetic Activity in Arabidopsis.” Plant Science 319: 111251. 35487661 10.1016/j.plantsci.2022.111251

[pbi70674-bib-0032] Sun, Q. , S. Jiang , T. Zhang , et al. 2019. “Apple Nac Transcription Factor Mdnac52 Regulates Biosynthesis of Anthocyanin and Proanthocyanidin Through Mdmyb9 and Mdmyb11.” Plant Science 289: 110286. 31623786 10.1016/j.plantsci.2019.110286

[pbi70674-bib-0033] Sun, W. , X. Meng , L. Liang , et al. 2017. “Overexpression of a Freesia Hybrida Flavonoid 3‐O‐Glycosyltransferase Gene, Fh3gt1, Enhances Transcription of Key Anthocyanin Genes and Accumulation of Anthocyanin and Flavonol in Transgenic Petunia ( *Petunia hybrida* ).” In Vitro Cellular & Developmental Biology. Plant 53: 478–488.

[pbi70674-bib-0034] Sun, W. , S. Sun , H. Xu , et al. 2022. “Characterization of Two Key Flavonoid 3‐O‐Glycosyltransferases Involved in the Formation of Flower Color in Rhododendron Delavayi.” Frontiers in Plant Science 13: 863482. 35651780 10.3389/fpls.2022.863482 PMC9149423

[pbi70674-bib-0035] Tafessu, A. , R. O'Hara , S. Martire , et al. 2023. “H3.3 Contributes to Chromatin Accessibility and Transcription Factor Binding at Promoter‐Proximal Regulatory Elements in Embryonic Stem Cells.” Genome Biology 24: 25. 36782260 10.1186/s13059-023-02867-3 PMC9926682

[pbi70674-bib-0036] Vogt, T. , and P. Jones . 2000. “Glycosyltransferases in Plant Natural Product Synthesis: Characterization of a Supergene Family.” Trends in Plant Science 5: 380–386. 10973093 10.1016/s1360-1385(00)01720-9

[pbi70674-bib-0037] Voos, W. , and K. Pollecker . 2020. “The Mitochondrial Lon Protease: Novel Functions Off the Beaten Track?” Biomolecules 10: 253. 32046155 10.3390/biom10020253 PMC7072132

[pbi70674-bib-0038] Wang, M. , Q. Shen , J. Pang , et al. 2024. “Study on Chemical Constituents and Antioxidant Activities of *Dianthus caryophyllus* L.” Frontiers in Plant Science 15: 1438967. 39239204 10.3389/fpls.2024.1438967 PMC11374617

[pbi70674-bib-0039] Wang, M. , R. Sun , J. Wang , et al. 2022. “Determination of Kaempferidetriglycoside in Diauthus by Hplc.” Chemical Research 33: 503–511.

[pbi70674-bib-0040] Wang, W. , W. Cai , J. Zhu , and Y. Zhu . 2025. “Alphafold 3‐Assisted Deciphering of the DNA Recognition by Dreb1 Transcription Factors in Rice.” International Journal of Molecular Sciences 26: 6395. 40650172 10.3390/ijms26136395 PMC12249877

[pbi70674-bib-0041] Yagi, M. , S. Kosugi , H. Hirakawa , et al. 2014. “Sequence Analysis of the Genome of Carnation ( *Dianthus caryophyllus* L.).” DNA Research 21: 231–241. 24344172 10.1093/dnares/dst053 PMC4060945

[pbi70674-bib-0042] Yuan, Y. , J. Zhang , J. Fan , et al. 2018. “Microwave Assisted Extraction of Phenolic Compounds From Four Economic Brown Macroalgae Species and Evaluation of Their Antioxidant Activities and Inhibitory Effects on Α‐Amylase, Α‐Glucosidase, Pancreatic Lipase and Tyrosinase.” Food Research International 113: 288–297. 30195523 10.1016/j.foodres.2018.07.021

[pbi70674-bib-0043] Zhang, D. , Z. Yao , B. Hou , W. Zhang , and L. Sun . 2017. “Inhibitory Effect of Extracts From Nymphaea Hybrid Flowers on Pancreatic Lipase.” Food Science and Technology 42: 227–231.

[pbi70674-bib-0044] Zhao, L. , J. Wang , Y. Zhou , et al. 2025. “Genome‐Wide Identification of Plant C2 Domain‐Containing Protein Family and the Role of Osntmc2t2.2 Under Chilling Stress in Rice.” Environmental and Experimental Botany 237: 106202.

[pbi70674-bib-0047] Zhao, T. , J. Lu , H. Zhang , et al. 2022. “Histone H3.3 Deposition in Seed Is Essential for the Post‐Embryonic Developmental Competence in Arabidopsis.” Nature Communications 13: 7728. 10.1038/s41467-022-35509-6 PMC9747979 36513677

[pbi70674-bib-0045] Zhong, C. , Y. Tang , B. Pang , et al. 2020. “The R2r3‐Myb Transcription Factor Ghmyb1a Regulates Flavonol and Anthocyanin Accumulation in Gerbera Hybrida.” Horticulture Research 7: 78. 32435501 10.1038/s41438-020-0296-2 PMC7237480

[pbi70674-bib-0046] Zhou, X. , S. Li , and X. Yang . 2022. “The Dcps1 Cooperates With Osdla During Pollen Development and 2n Gamete Production in Carnation Meiosis.” BMC Plant Biology 22: 259. 35610560 10.1186/s12870-022-03648-z PMC9128087

