# Supplementary material for: DcH3.3 and DcNAC1 Regulate the Expression of UGT73A93 Involved in the Changes in Flower Colour and Fungal Resistance in Carnation
Source: Plant Biotechnol J. 2026 Apr 25;24(8):4863–77. doi: 10.1111/pbi.70674 (PMC13387883; doi:10.1111/pbi.70674)
Supplement: Supplementary file 1 — Figure S1: Functional characterization of UGT73A93 (DcGT4) gene following its overexpression in T3 generation transgenic tobacco lines (T1). (A) Differences in colour between wild‐type (WT), empty vector (EV) and transgenic tobacco flowers. (B) The values of L*, a* and b* classified by the CIELAB systems. (C–E) Expression of UGT73A93 normalised to NbGAPDH gene in WT, EV and transgenic lines of stem (C), leaf (D) and flower (E). (F‐H) Contents of the total flavonoids in stem (F), leaf (G) and flower (H). (I) Contents of the total anthocyanins in flower. (J) HPLC chromatograms of flavonols glucosylated in vitro. (K, L) Contents of Kaempferol 3‐O‐sophoroside and Kaempferol 3‐O‐rutinoside in flower. Error bars represent ± SD from three replicates. Asterisks indicate significant differences by multiple t‐test (ns, p > 0.05; *p < 0.05; **p < 0.01; ***p < 0.001; ****p < 0.0001). Scale bars represent 1 cm. Figure S2: Ferric ion reducing antioxidant power (FRAP) of stem (A), leaf (B) and flower (C) extraction in T1 generation transgenic tobacco. Figure S3: Ferric ion reducing antioxidant power (FRAP) of stem (A), leaf (B) and flower (C) extraction in T3 generation transgenic tobacco. Figure S4: Expression profiles of flavonoid‐related biosynthetic genes in flowers of transgenic carnation lines carrying UGT73A93 (DcGT4) genes. The expression levels of DcFLS (A), DcGT5 (B), DcF3′H (C), DcDFR (D) gene in carnation stems, leave and flower. FLS, flavonol synthase; F3′H, flavonoid 3′‐hydroxylase; DFR, dihydroffavonol 4‐reductase; GT5, UDP‐glycose flavonoid glycosyltransferase 5. Error bars in represent ± SEM from three replicates. Asterisks indicate significant differences by multiple t‐test (ns, p > 0.05; *p < 0.05; **p < 0.01; ***p < 0.001; ****p < 0.0001). Figure S5: Ferric ion reducing antioxidant power (FRAP) of stem (A), leaf (B) and flower (C) extraction in carnation. [file PBI-24-4863-s001.docx]

**FIGURE S1** Functional characterization of *UGT73A93* (*DcGT4*) gene following its overexpression in T3 generation transgenic tobacco lines (T1). (A) Differences in color between wild-type (WT), empty vector (EV), and transgenic tobacco flowers. (B) The values of L*, a* and b* classified by the CIELAB systems. (C-E) Expression of *UGT73A93* normalized to *NbGAPDH* gene in WT, EV and transgenic lines of stem (C), leaf (D), and flower (E). (F-H) Contents of the total flavonoids in stem (F), leaf (G) and flower (H). (I) Contents of the total anthocyanins in flower. (J) HPLC chromatograms of flavonols glucosylated in vitro. (K-L) Contents of Kaempferol 3-*O*-sophoroside and Kaempferol 3-*O*-rutinoside in flower. Error bars represent ± SD from three replicates. Asterisks indicate significant differences by multiple t-test (ns, P>0.05; *, P < 0.05; **, P < 0.01; ***, P < 0.001; ****, P < 0.0001). Scale bars represent 1 cm.

**FIGURE S2** Ferric ion reducing antioxidant power (FRAP) of stem (A), leaf (B), and flower (C) extraction in T1 generation transgenic tobacco.

**FIGURE S3** Ferric ion reducing antioxidant power (FRAP) of stem (A), leaf (B), and flower (C) extraction in T3 generation transgenic tobacco.

**FIGURE S4** Expression profiles of flavonoid-related biosynthetic genes in flowers of transgenic carnation lines carrying *UGT73A93* (*DcGT4*) genes. The expression levels of *DcFLS* (A), *DcGT5* (B), *DcF3’H* (C), *DcDFR* (D) gene in carnation stems, leave and flower. FLS, flavonol synthase; F3’H, flavonoid 3′- hydroxylase; DFR, dihydroffavonol 4-reductase; GT5, UDP-glycose flavonoid glycosyltransferase 5. Error bars in represent ± SEM from three replicates. Asterisks indicate significant differences by multiple t-test (ns, P>0.05; *, P < 0.05; **, P < 0.01; ***, P < 0.001; ****, P < 0.0001).

**FIGURE S5** Ferric ion reducing antioxidant power (FRAP) of stem (A), leaf (B), and flower (C) extraction in carnation.
